# FANTOM5 CAGE profiles of human and mouse reprocessed for GRCh38 and GRCm38 genome assemblies

**DOI:** 10.1038/sdata.2017.107

**Published:** 2017-08-29

**Authors:** Imad Abugessaisa, Shuhei Noguchi, Akira Hasegawa, Jayson Harshbarger, Atsushi Kondo, Marina Lizio, Jessica Severin, Piero Carninci, Hideya Kawaji, Takeya Kasukawa

**Affiliations:** 1 RIKEN Center for Life Science Technologies, Division of Genomic Technologies, 1-7-22 Suehiro-cho, Tsurumi-ku, Yokohama, Kanagawa 230-0045, Japan; 2 RIKEN Omics Science Center, 1-7-22 Suehiro-cho, Tsurumi-ku, Yokohama, Kanagawa 230-0045, Japan; 3 RIKEN Preventive Medicine and Diagnosis Innovation Program, 2-1 Hirosawa, Wako, Saitama 351-0198, Japan; 4 RIKEN Advanced Center for Computing and Communication, Preventive Medicine and Applied Genomics Unit, 1-7-22 Suehiro-cho, Tsurumi-ku, Yokohama, Kanagawa 230-0045, Japan

**Keywords:** Data processing, Gene expression

## Abstract

The FANTOM5 consortium described the promoter-level expression atlas of human and mouse by using CAGE (Cap Analysis of Gene Expression) with single molecule sequencing. In the original publications, GRCh37/hg19 and NCBI37/mm9 assemblies were used as the reference genomes of human and mouse respectively; later, the Genome Reference Consortium released newer genome assemblies GRCh38/hg38 and GRCm38/mm10. To increase the utility of the atlas in forthcoming researches, we reprocessed the data to make them available on the recent genome assemblies. The data include observed frequencies of transcription starting sites (TSSs) based on the realignment of CAGE reads, and TSS peaks that are converted from those based on the previous reference. Annotations of the peak names were also updated based on the latest public databases. The reprocessed results enable us to examine frequencies of transcription initiations on the recent genome assemblies and to refer promoters with updated information across the genome assemblies consistently.

## Background & Summary

A complete genome sequence provides an essential infrastructure to study an organism at the molecular level. It does not only represent an entire set of genetically inherited information, but also a coordinated system to describe genomic entities, such as nucleotide polymorphisms, genes, and regulatory elements. The representation of genome entities along a genomic reference is termed genome annotation^1^. The Genome Reference Consortium has been providing a variety of reference genome assemblies, updated in a timely manner in order to reflect the outcomes of recent research^2^. The latest assemblies available for human and mouse are versioned as GRCh38/hg38 and GRCm38/mm10. Recent efforts to improve the genome annotations based on the current assemblies were consequently carried, including the update of RefSeq^3^ and GENCODE transcripts^4^.

The FANTOM (Functional ANnoTation Of Mammalian genomes) project was launched to provide functional annotation for mammalian genomes, in particular focusing on transcriptome profiles^5^. The fifth edition of the FANTOM project (FANTOM5) profiled genome-wide transcription start sites (TSSs) in mammalian genomes by using CAGE (Cap Analysis of Gene Expression) with single-molecule sequencers (HeliScope); it then revealed a promoter-level expression atlas across mammalian primary cells, tissues, cell lines and time course samples, as well as a transcribed enhancer atlas^6–8^. In the CAGE method, cDNA fragments with a cap structure at their 5′-end are sequenced and the sequenced reads are aligned to a reference genome to count frequencies of transcription initiation. The resulting profiles represent activities of transcription initiation (TSS activities) at a single base-pair resolution across the entire genome^9^. These profiles were then used to define TSS peaks representative of promoters. Because the reference genome assemblies are universal and publicly shared, TSS activities can be measured on a common coordinate system.

In the original publications of the FANTOM5 papers, the GRCh37/hg19 human and NCBI37/mm9 mouse genome assemblies were used. Here we reprocessed the FANTOM5 data to make it available on the current assemblies GRCh38/hg38 and GRCm38/mm10. We remapped individual CAGE reads, to fully benefit of improvements such as correction of erroneous genome sequences. In contrast, we converted the genomic coordinates of the CAGE peaks into the current assemblies by liftOver tool^10^, so that we can easily make corresponding between the reprocessed CAGE peaks and the existing FANTOM5 resources across the assemblies. Following the conversion, we added new CAGE peaks introduced in the latest genome assemblies, for example, ones for newly introduced genes. For this purpose, we used the result of peak-calling by the same method as reported in the original report^8^ based on the realigned CAGE reads with the latest genome assemblies (Data Citation 1), and chose non-overlapped CAGE peaks in the result to merged with the converted CAGE peaks.

**Figure 1:**
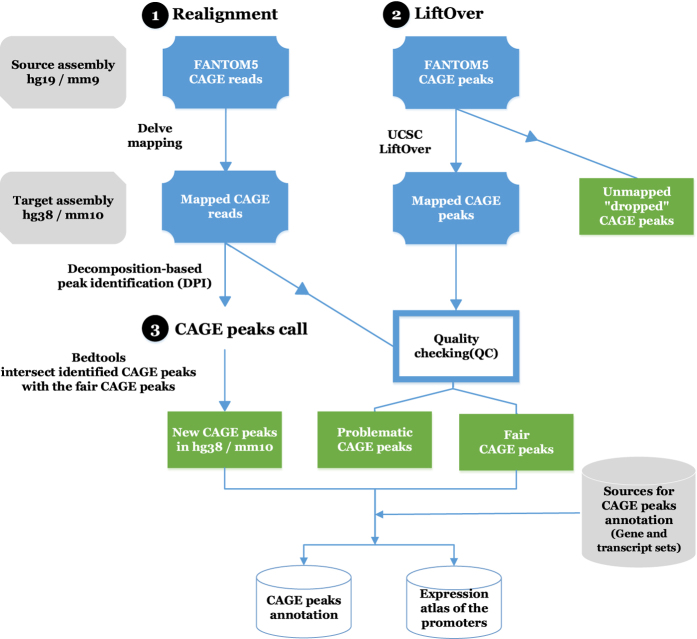
Work flow of FANTOM5 data re-processing. The figure describes the reprocessing of the FANTOM5 data. The workflow encompasses three processes; CAGE reads realignment (1), CAGE peaks liftOver (2) and CAGE peaks call (3). The source datasets are in (GRCH37/hg19) and (NCBI37/mm9). The target assembly is (GRCH38/hg38) and (GRCm38/mm10). CAGE reads realignment result in mapped CAGE peaks, CAGE peaks liftOver result in two sets of CAGE peaks (mapped and unmapped). And the CAGE peaks call result in new CAGE peaks in the latest genomes. Process (1) and (2) are followed by quality checking (QC). The QC filtered the mapped CAGE peaks into fair and problematic CAGE peaks. The set of problematic and dropped CAGE peak regions are investigated and manually curated. The new CAGE peaks from (3) are intersected with the fair CAGE peaks using bedtools (intersectbed) to define non-overlapped CAGE peaks (new CAGE peaks). The fair and new CAGE peaks are annotated with the latest gene and transcript models and their expression tables are calculated.

Unique identifiers were assigned to the CAGE peaks to provide the consistent reference to the CAGE peaks even after future updates. Majority of the peaks were successfully converted, and a few unexpected conversions were manually corrected. As a consequence, expression and gene annotations of the CAGE peaks were updated (Fig. 1). The reprocessed data of the FANTOM5 human and mouse CAGE datasets (Data Citations 2,3,4,5,6,7,8,10) are publicly available from the FANTOM5 data web site (http://fantom.gsc.riken.jp/5/datafiles/reprocessed/), LSDB Archive (Data Citation 11) and Figshare (hg38 (Data Citation 12) and mm10 (Data Citation 13)).

## Methods

### Realignment of CAGE reads

The FANTOM5 CAGE reads (Data Citations 2,3,4,5,6,7,8,10) were realigned by Delve version 0.95 with the GRCh38/hg38 and GRCm38/mm10 genome assemblies following the same procedure to the previous report^6–8,11^. The genome assembly files (FASTA format) were downloaded from the UCSC genome browser database^12^, in particular the files under ‘bigZips’ directories ((Data Citation 14) for human and (Data Citation 15) for mouse) were used and only primary chromosomes (chr1..22, X, Y, M for human and chr1..19, X, Y, M for mouse) were considered as a reference in the realignment. The same criteria detailed in the original report^8^, mapping quality >20 and sequence identity 85%, were employed for selecting successful alignments. The former threshold indicates that CAGE reads were discarded if they were aligned with two or more loci and it was difficult to determine the originating locus uniquely.

### Conversion of the CAGE peaks genomic coordinates

The genomic coordinates of the original CAGE peaks (hg19 (Data Citation 16) and mm9 (Data Citation 17); or (Data Citation 11)) were computationally converted to the latest genome assemblies, followed by manual curation (Fig. 1). First we applied a software utility to convert between genomes, liftOver^10^, with option --minMatch=1 and the chain files from hg19 to hg38 (Data Citation 18) and from mm9 to mm10 (Data Citation 19). A few of the CAGE peaks couldn’t be located in the latest genome assemblies (termed ‘dropped’), where the ratios of dropped peaks were less than 0.1% (Table 1).

Next we examined whether the mapped peaks were supported by the presence of any realigned CAGE reads, to confirm that the converted locations still have experimental evidences. We found that 335 and 76 peaks for human and mouse respectively had no support, and excluded them from downstream analysis by classifying them as ‘problematic’. We also examined internal overlap within the converted peaks, since they are not supposed overlap each other, based on their definition^8^. We found ten peaks overlapping in four loci only on hg38, which were caused by the fusion of originally distinct genomic regions or by unintentional stretch of a single CAGE peak after the conversion. We excluded four peaks, one peak for each overlapping locus, and classified them as ‘problematic’ as well (Table 2).

Lastly we examined whether the length of CAGE peaks was altered after the conversion to the latest genomic coordinates. We found eight peaks whose lengths were changed more than one base due to insertions and/or deletions in the updated genome assemblies. We manually updated the genomic coordinates of two out of eight CAGE peaks, to make them consistent with the CAGE read realignments (Table 2). As a result, we successfully generated a clean set of CAGE peaks (called ‘fair’) on the latest genome assemblies, representing more than 99% of the original ones (Table 1).

### Integration of newly identified CAGE peaks in the latest genome assemblies

We complemented the converted CAGE peaks with a new set of CAGE peaks identified to the same method to the previous study (DPI, decomposition-based peak identification)^8^ based on the realigned CAGE reads with the latest genome assemblies (Data Citation 1). To identify their overlaps, we used intersectBed command with option -s from Bedtools^13^. Non-overlapped CAGE peaks identified by DPI were merged with the converted CAGE.

### Assignment of accession numbers to the CAGE peaks

Identifiers assigned to the CAGE peaks (CAGE peak ID) were formatted based on their genomic coordinates (Table 3). To have a consistent way to refer individual peaks, we assigned accessions to them, in the form of ‘hg_(serial_num).(version_num)’ and ‘mm_(serial_num).(version_num)’ for human and mouse, respectively. This enabled us to refer them independently of their genome assemblies and their coordinates, with explicit indication of versions. For backward traceability, the new ID is concatenated with the original ID by semicolon (;) in the final files. To avoid confusion of genome versions, we added ‘hg19’ or ‘mm9’ as prefix of the original ID.

### Re-annotation of CAGE peaks with the latest gene and protein databases

To take advantage of recent genome annotations, in particular long-noncoding RNAs^5^, we updated the association of the CAGE peaks with genes, transcripts, and proteins. The process consists of the two steps: (i) associate CAGE peaks to transcripts, and subsequently to genes and proteins (ii) assign human-readable short names. The first step, association with transcripts, was achieved by finding the TSS of transcripts within 500 bp flanking region of the CAGE peak (flanking regions are limited to 50 bp for 5′-end not derived from transcription initiation by RNA Polymerase II, such as small nucleolar RNA), choosing the nearest transcripts, and associating CAGE peaks to gene and protein models based on the nearest transcripts. In the second step, human-readable short names were assigned in the same form to the original ones, ‘p<serial>**@**<HGNC/MGI/Entrez Gene name>’. Serial numbers were chosen not to conflict with already existing names, so that all names are unique not only within the current genome assembly but also across genome assemblies of a species.

We used the following annotation databases for re-annotation as of March 31, 2016: GENCODE^4^, Entrez Gene^14^, HUGO Gene Nomenclature Committee (HGNC) database^15^, the Mouse Genome Database (MGD)^16^ and the UCSC Genome Browser^12^. Transcript sets are from GENCODE (human/mouse), RefSeq (human/mouse), UCSC genes (human/mouse) and mRNAs in the UCSC genome browser (human/mouse). Gene models used for the annotation are from HGNC (human), MGI (mouse) and Entrez Gene (human/mouse). The protein sets are from UniProt (human/mouse).

### Recalculation of promoter-level expression tables

As in the previous report^8^, we counted the read counts under the CAGE peaks on the current genome assemblies. The counts were normalized as TPM (tags per million) after scaling by normalization factors calculated by RLE (Relative Log Expression) method^17,18^.

### Code availability

1. The latest version of the source code of the Delve mapper is openly available from http://fantom.gsc.riken.jp/5/suppl/delve/delve-0.95.tgz.
2. The latest version of the source code of the DPI is openly available from https://github.com/hkawaji/dpi1.

## Data Records

The original data of raw reads (CAGE tags) for human and mouse is available from the DDBJ Sequence Read Archive (DRA) (Data Citations 2,3,4,5,6,7,8,10). The human reprocessed dataset is available at (Data Citation 12) and mouse reprocessed dataset is available at (Data Citation 13). The results of realignment of CAGE reads in BAM format (.bam) with their index files (.bai) are located under ‘basic/*’ directories at http://fantom.gsc.riken.jp/5/datafiles/reprocessed/ or in LSDB Archive (Data Citation 11). The reprocessed CTSS file of each sample in BED format is also located in the same directory.

The genomic coordinates of the reprocessed CAGE peak regions in the BED format are in (Data Citations 11,12,13). The tab-delimited annotation files of CAGE peaks are in (Data Citations 11,12,13). The expression profiles of CAGE peaks in OSC table (Order Switchable Column table) (https://sourceforge.net/projects/osctf/) can be found in (Data Citations 11,12,13).

Sample metadata is stored in sample and data relationship format (SDRF)^19^ at the ‘basic/’ directory at http://fantom.gsc.riken.jp/5/datafiles/reprocessed/.

## Technical Validation

### Number of realigned CAGE reads

The numbers of CAGE reads successfully aligned to the genome assemblies are shown in (Table 4). Slightly less number of CAGE reads were successfully aligned with the current genome assemblies than the original ones for both human and mouse. This can be explained by the incorporation of complex and duplicated regions into the current genome assemblies, which made uniquely aligned CAGE reads in the original assemblies as aligned with multiple locations in the current assemblies. Overall, very similar number of CAGE reads are available before and after the realignments.

### Number of converted CAGE peaks

The numbers of CAGE peaks converted from the original assemblies are shown in (Table 1). The majority of the peaks, more than 99%, were classified into the category ‘fair’. The detailed numbers of problematic and dropped peaks are shown in (Tables 5,6,7,8). We further inspected the problematic CAGE peaks, and found that the CAGE reads corresponding to the original peak were discarded due to low mapping quality (that is, aligned to multiple regions). This is again likely due to duplicated genomic regions that were introduced in hg38 and mm10.

### Comparison of the fair CAGE peaks and DPI-called CAGE peaks

We compared the converted CAGE peaks in the ‘fair’ category with the peaks identified based on the read realignments with the latest genome assemblies (DPI CAGE peaks). The number of their overlaps (Table 9) shows that approximately 95% of a peak set is covered by the other. This result indicates high concordance of the converted CAGE peaks to the ones based on realignments, and underline a certain amount of additional CAGE peaks, ~8,600 and ~5,700 in human and mouse, were newly included in the CAGE peak set provided here.

### Expressions of the CAGE peaks

**Figure 2:**
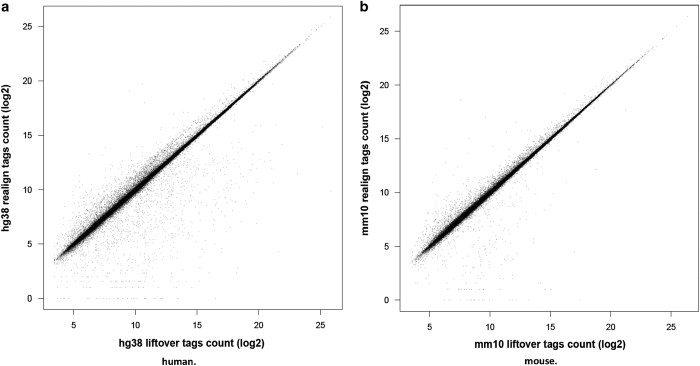
Correlation between the CAGE tags count of the aligned CAGE reads and the liftOver CAGE peaks. The scatterplot shows the correlation between the number of tag count within the regions of aligned CAGE reads and the liftOver CAGE peaks. [2a] human and [2b] mouse.

Next, we examined expressions of the CAGE peaks on the original genome assemblies and the current ones (Fig. 2). Their expression levels are tightly consistent across the assemblies, as correlation coefficients are more than 0.99, using Spearman’s and Pearson’s correlation for human and mouse. At the same time, the scatter plot indicates a limited number of peaks that were quantified differently. For the majority of those inconsistent peaks, the expression levels were low, very likely due to the incorporation of duplicated regions in the current assemblies.

### Annotation of the CAGE peaks

We compared CAGE peak annotations based on the latest genomes (hg38/mm10) with the original ones based on hg19/mm9. First, we compared the number of CAGE peaks that was associated with any transcripts, genes in Entrez Gene (human and mouse), HGNC (human), and proteins in UniProt (Table 10). The numbers of associated UniProt proteins were almost similar. However, the number of associated transcripts and genes were increased from the original ones by 15–20%. This may reflect the growing pace of the gene annotations in public databases, in particular non-coding RNAs and complex isoforms of protein coding genes. Next, we compared the number of CAGE peaks classified based on gene categories as defined in Entrez Genes (Table 11). The number of CAGE peaks with protein coding genes increased in the same ratio as with all genes. On the other hand, CAGE peaks with non-coding genes were drastically increased, which may reflect the recent numerous findings in non-coding RNA studies^5^.

## Usage Notes

In addition to the data files of realignments and converted CAGE peaks described in data citation, the data are made accessible in TET (Table Extraction Tool)^20^, SSTAR (Semantic catalog of Samples, Transcription initiation And Regulators)^21^, ZENBU^22^, and UCSC genome browser via data hub^23^.

## Additional Information

**How to cite this article**: Abugessaisa, I. *et al.* FANTOM5 CAGE profiles of human and mouse reprocessed for GRCh38 and GRCm38 genome assemblies. *Sci. Data* 4:170107 doi: 10.1038/sdata.2017.107 (2017).

**Publisher**’**s note**: Springer Nature remains neutral with regard to jurisdictional claims in published maps and institutional affiliations.

## Supplementary Material



## Figures and Tables

**Table 1:** CAGE peaks counts.

**CAGE peak category**	**Human**	**Ratio**	**Mouse**	**Ratio**
Fair	201,295	99.75%	158,878	99.94%
Problematic	339	0.17%	76	0.05%
Dropped	168	0.08%	12	0.01%
Total	201,802	—	158,966	—
Categories of the CAGE peaks converted to the current genome assemblies.				

**Table 2:** Manual correction of the genomic coordinates.

**CAGE peaks**	**Issues**	**Workaround**	**Note**
hg19::chr1:145176389..145176406,+;hg_14198.1	overlapping (1st group)/changing length	kept in 'problematic'	dut to the unintentional conversion of the CAGE peak
hg19::chr1:146369648..146369656,−;hg_14199.1	overlapping (1st group)	resecued to 'fair'	
hg19::chr1:146544055..146544062,−;hg_14200.1	overlapping (1st group)	resecued to 'fair'	
hg19::chr1:146556295..146556310,−;hg_14201.1	overlapping (1st group)	resecued to 'fair'	
hg19::chr1:120905986..120906002,+;hg_14114.1	overlapping (2nd group)	resecued to 'fair'	due to the merge of two genes in hg38, chose the longer peak
hg19::chr1:149399224..149399230,−;hg_14115.1	overlapping (2nd group)	kept in 'problematic'	
hg19::chr1:120838328..120838358,−;hg_4940.1	overlapping (3rd group)	kept in 'problematic'	
hg19::chr1:143913790..143913841,+;hg_4941.1	overlapping (3rd group)	resecued to 'fair'	due to the merge of two genes in hg38, chose the longer peak
hg19::chrX:52112158..52112165,+;hg_196395.1	overlapping (4th group)	kept in 'problematic'	
hg19::chrX:52386980..52387013,−;hg_196396.1	overlapping (4th group)	resecued to 'fair'	due to the merge of two regions of single genes in hg38, chose the longer peak
hg19::chr3:124646690..124646794,−;hg_44259.1	changing length	kept as is	
hg19::chr7:101930149..101930221,+;hg_80245.1	changing length	kept as is	
hg19::chr8:143857402..143857446,−;hg_95429.1	changing length	kept as is	
hg19::chr10:61122262..61122358,−;hg_109451.1	changing length	kept as is	
hg19::chr14:22689737..22689741,−;hg_142211.1	changing length	changed CAGE peak regions	inserted 3 'T' nucleotides at the start of CAGE peaks, which would be removed
hg19::chr17:26684480..26684571,−;hg_164987.1	changing length	kept as is	
hg19::chrX:114690490..114690493,−;hg_200246.1	changing length	changed CAGE peak regions	inserted 17 'T' nucleotides at the start of CAGE peaks, which would be removed
hg19::chrX:148713374..148713438,−;hg_200825.1	changing length	kept as is	
This table list the CAGE peaks and the issues detected during genomic coordinates conversion. It shows the workaround solution to each issue and additional notes.			

**Table 3:** The naming scheme of the CAGE peaks before and after reprocessing.

	**Original genome assembly**	**Latest genome assembly**
Genomic coordinate	From 564639 bp to 564649 bp* of chromosome 1 on the forward strand, based on the genome assmbly hg19	From 629259 bp to 629269 bp* of chromosome 1 on the forward strand, based on the genome assembly hg38
CAGE peak ID	chr1:564639..564649,+	hg19::chr1:564639..564649,+;hg_2.1
Accession	—	hg_2.1
Short Description	p3@MTND1P23	p3@MTND1P23
Full description	CAGE_peak_3_at_MTND1P23_5end	—
The table shows the naming rules of the CAGE peaks used in the published FANTOM5 human and mouse dataset and the newly assigned CAGE peaks ID after liftOver.		

*These positions are based on the coordinates in BED format

**Table 4:** CAGE read counts.

**Species**	**Genome assemblies**	**Successfully aligned CAGE reads**	**Ratio to the original assembly**	**Successful alignments starting from the peaks**	**Ratio to the original assembly**
Human	GRCh37/hg19	7,002,308,021	—	5,288,118,024	—
	GRCh38/hg38	6,846,664,897	97.8%	5,158,308,820	97.5%
Mouse	NCBI37/mm9	4,694,137,744	—	3,491,906,982	—
	GRCm38/mm10	4,687,916,697	99.9%	3,509,420,580	100.5%
The CAGE read counts successfully aligned with the genome assemblies and within the CAGE peaks.					

**Table 5:** The problematic peaks in hg38.

**Chromosome**	**Number of problematic regions**
chr1	118
chr2	14
chr3	2
chr4	6
chr5	2
chr6	27
chr7	13
chr8	1
chr9	7
chr10	8
chr11	8
chr12	4
chr13	1
chr14	4
chr15	5
chr16	8
chr17	7
chr18	5
chr19	1
chr21	90
chrX	5
chrY	3
Total	339
Table list total number of problematic CAGE peak regions per each chromosome in hg38.	

**Table 6:** The problematic peaks in mm10.

**Chromosome**	**Number of problematic regions**
chr3	1
chr4	1
chr5	3
chr9	3
chr13	1
chr15	1
chr16	2
Total	12
Table list total number of problematic CAGE peak regions per each chromosome in mm10.	

**Table 7:** The dropped peaks from hg19.

**Chromosome**	**Number of dropped peaks**
chr1	14
chr2	2
chr3	1
chr6	2
chr7	105
chr8	3
chr11	1
chr14	8
chr17	2
chr19	9
chr22	8
chrM	8
chrX	5
Total	168
Table list total number of dropped CAGE peak regions during genomic coordinates conversion per each chromosome in hg19.	

**Table 8:** The dropped peaks from mm9.

**Chromosome**	**Number of dropped peaks**
chr1	7
chr2	1
chr3	1
chr4	5
chr5	3
chr7	2
chr8	10
chr10	2
chr12	2
chr13	1
chr14	25
chr16	1
chrX	6
chrY	10
Total	76
Table list total number of dropped CAGE peak regions during genomic coordinates conversion per each chromosome in mm10.	

**Table 9:** Number of new CAGE peaks identified by peaks calling and their overlap with the converted CAGE peaks.

**Species**	**Converted ‘Fair’ CAGE peaks**	**CAGE peaks based on the realignments**
*Human*		
All peaks	201,295	195,444
Overlapped peaks with the other dataset	189,679 (94.2%)	186,828 (95.6%)
Non-oeverlapped peaks with the other dataset	11,616 (5.8%)	8,616 (4.4%)
*Mouse*		
All CAGE peaks	158,878	155,006
Overlapped with the other dataset	152,189 (95.8%)	149,212 (96.3%)
Non-oeverlapped with the other dataset	6,689 (4.2%)	5,794 (3.7%)
The table shows the total number and the ratio of the overlapped and non-overlapped CAGE peaks between the converted ‘fair’ CAGE peaks and the new CAGE peaks identified by the decomposition-based peak identification (DPI).		

**Table 10:** Counts of CAGE peaks associated with transcripts, genes and proteins.

**Species**	**Genome assemblies**	**Associated transcripts**	**Associated Protein (UniProt)**	**Associated HGNC**	**Associated MGI**	**Associated Enterz Gene**
Human	GRCh37/hg19	93,558	56,011	82,257	—	82,150
	GRCh38/hg38	108,791	57,935	96,998	—	97,560
Mouse	NCBI37/mm9	61,072	47,755	—	—	56,744
	GRCm38/mm10	89,471	47,657	—	84,308	79,319
The table shows the number of (robust) CAGE peaks associated with known transcripts, genes in Entrez Gene, HGNC and MGI, and proteins in UniProt. The numbers in GRCh38/hg38 and GRCm37/mm10 rows were counted by the reprocessing project. The numbers of GRCh37/hg19 and NCBI37/mm9 were retrieved from the original paper.						

**Table 11:** Number of peaks associated with Entrez Gene categories.

**Species**	**Genome assemblies**	**Protein-coding**	**Pseudogene**	**miscRNA**/**miRNA**	**snRNA**/**snoRNA**	**other**/**unknown**
human	GRCh37/hg19	79,735	489	1,755	126	163
	GRCh38/hg38	90,351	737	5,731	124	300
mouse	NCBI37/mm9	55,217	435	1,356	22	16
	GRCm38/mm10	77,224	208	3,156	16	934
The table shows the number of (robust) CAGE peaks associated with Entrez Gene categories. The numbers in GRCh38/hg38 and GRCm37/mm10 rows were counted by the reprocessing project. The numbers of GRCh37/hg19 and NCBI37/mm9 were retrieved from the original paper.						
